# Diagnosis of primary ovarian cancer

**DOI:** 10.4103/0974-1208.69336

**Published:** 2010

**Authors:** Viroj Wiwanitkit

**Affiliations:** Department of Laboratory Medicine, Faculty of Medicine, Chulalongkorn University, Bangkok - 10330, Thailand

Sir,

I read the recent rare case report on primary ovarian cancer by Panda *et al*. with a great interest.[1] According to this report, some critical concerns on the diagnosis of primary ovarian cancer should be mentioned. Panda *et al*. made a conclusion that “now, with ultrasonographic advances, it can be diagnosed early, leading to conservative treatment and preservative surgery.”[1] Indeed, the role of ultrasonography for the detection of ectopic pregnancy is of no doubt. However, its role for specific diagnosis of primary ovarian pregnancy is still questionable. Only a gestation sac in adnexa area, which cannot definitely diagnose of primary ovarian pregnancy, is the common ultrasonographic finding.[2] The actual diagnosis is usually due to the confirmation by histopathological study. The ultrasonography could only help early detect the ectopic pregnancies, of which, only a few can be primary ovarian pregnancy.

## References

[1] Panda S, Darlong LM, Singh S, Borah T (2009). Case report of a primary ovarian pregnancy in a primigravida. J Hum Reprod Sci.

[2] Chandrasekhar C (2008). Ectopic pregnancy: A pictorial review. Clin Imaging.

